# Predicting Optimal Combination LT4 + LT3 Therapy for Hypothyroidism Based on Residual Thyroid Function

**DOI:** 10.3389/fendo.2019.00746

**Published:** 2019-11-15

**Authors:** Joseph DiStefano, Jacqueline Jonklaas

**Affiliations:** ^1^Departments of Computer Science and Medicine, University of California, Los Angeles, Los Angeles, CA, United States; ^2^Division of Endocrinology, Georgetown University, Washington, DC, United States

**Keywords:** simulation, combination therapy, levothyroxine, liothyronine, residual thyroid function, hypothyroidism etiology

## Abstract

**Objective:** To gain insight into the mixed results of reported combination therapy studies conducted with levothyroxine (LT4) and liothyronine (LT3) between 1999 and 2016.

**Methods:** We defined trial success as improved clinical outcome measures and/or patient preference for added LT3. We hypothesized that success depends strongly on residual thyroid function (RTF) as well as the LT3 added to sufficient LT4 dosing to normalize serum T4 and TSH, all rendering T3 levels to at least middle-normal range. The THYROSIM app was used to simulate “what-if” experiments in patients and study designs corresponding to the study trials. The app graphically provided serum total (T4) and free (FT4) thyroxine, total (T3) and free (FT3) triiodothyronine, and TSH responses over time, to different simulated LT4 and combination LT4 + LT3 dosage inputs in patients with primary hypothyroidism. We compared simulation results with available study response data, computed RTF values that matched the data, classified and compared them with trial success measures, and also generated nomograms for optimizing dosages based on RTF estimates.

**Results:** Simulation results generated three categories of patients with different RTFs and T3 and T4 levels at trial endpoints. Four trial groups had >20%, four <10%, and five 10–20% RTF. Four trials were predicted to achieve high, seven medium, and two low T3 levels. From these attributes, we were able to correctly predict 12 of 13 trials deemed successful or not. We generated an algorithm for optimizing dosage combinations suitable for different RTF categories, with the goal of achieving mid-range normal T4, T3 and TSH levels. RTF is estimated from TSH, T4 or T3 measurements prior to any hormone therapy treatment, using three new nonlinear nomograms for computing RTFs from these measurements. Recommended once-daily starting doses are: 100 μg LT4 + 10–12.5 μg LT3; 100 μg LT4 + 7.5–10 μg LT3; and 87.5 μg LT4 + 7.5 μg LT3; for <10%, 10–20%, and >20% RTF, respectively.

**Conclusion:** Unmeasured and variable RTF is a complicating factor in assessing effectiveness of combination LT4 + T3 therapy. We have estimated and partially validated RTFs for most existing trial data, using THYROSIM, and provided an algorithm for estimating RTF from accessible data, and optimizing patient dosing of LT4 + LT3 combinations for future combination therapy trials.

## Introduction

Combination therapy for hypothyroidism using both levothyroxine (LT4) and liothyronine (LT3) continues to be a topic of much interest to physicians and patients alike (1–4). This interest has been spurred, in part, by the well-documented finding that the ratio of total thyroxine (T4) to total triiodothyronine (T3) increases during LT4 therapy, compared with endogenous euthyroidism (5), and also that T3 levels may be lower than in the native state (6). Furthermore, animal studies suggest T3 deficiency at the tissue level with LT4 therapy alone (7, 8). This interest persists despite the generally mixed results of combination therapy trials, with most results not demonstrating a benefit of such therapy in terms of improvement in quality of life, mood, or neurocognitive function, but some patients expressing preference for therapy containing LT3 (9–22). When examining outcomes of either quality of life, mood, or neurocognitive function, trials fall into 3 broad categories: those showing substantial clinical benefit of combination therapy (11, 16), those showing partial benefit based on some outcome measures, but not others (10, 13, 18, 21), and those showing no benefit (9, 12, 14, 15, 17, 19, 20, 22). Similarly, the seven trials that examined patient preference for combination therapy can be divided into two groups: those in which patients preferred the LT3-containing therapy (9–11, 13, 16), and those in which there was no preference (18, 22).

Numerous suggestions have been offered for why these combination therapy trials did not provide evidence of clinical benefits or greater patient preference. In addition to non-physiologic thyroid hormone ratios, potential shortcomings include use of once daily LT3 therapy rather than two or three times a day therapy, or short duration trials or underpowered trials (23). Examining these trials aggregated into meta-analyses (24–26) also has not revealed benefits of combination therapy, perhaps due in part to the heterogeneity of the trial populations and methods, which include different doses of LT4 and LT3 employed, etiology of hypothyroidism, unknown degree of residual thyroid function (RTF), treatment duration, different thyrotropin (TSH), free or total T4 and T3 levels achieved in the two groups, and the outcome measures employed (23). The current work is focused primarily on degree of residual thyroid function, which we postulate may be responsible for generating quite variable responses to and perceived effects of added exogenous LT3.

The THYROSIM app (27) is a freely accessible, well-validated and mechanistically-based simulator of human thyroid hormone and TSH regulation dynamics, developed and implemented as a facile web-based and personal device application. THYROSIM has a simple and intuitive user interface for teaching and conducting simulated “what-if” experiments, graphically providing temporal dynamic responses—namely levels of serum total T4, T3, free T4 (FT4), and free T3 (FT3), as well as TSH responses over time, to various simulated system and input perturbations in 70 kg humans (28, 29). It has also been modified to predict LT4 and LT3 replacement in pediatric patients (30), used to explore TSH dynamics in primary and secondary hypothyroidism (31), and applied to LT4 bioequivalence studies (29, 32). Furthermore, the utility of the app in clinical research also has been demonstrated more recently by predicting the potentially pathophysiological effects of over-the-counter thyroid supplements (33).

In order to gain insight into the mixed results of the 14 combination therapy trials, we developed the following two hypotheses to test predictively using the THYROSIM app and retrospectively using data from the trials. For both hypotheses, combination therapy is understood to mean addition of LT3 to LT4 dosing; and “success” of combination therapy was defined as benefit in terms of improved clinical outcome measures (quality of life, mood, or neurocognitive function) or patient preference for the added LT3.

### Working Hypothesis 1

Success with combination therapy will be greatest when the daily LT4 dose fraction is sufficient to normalize serum TSH and T4 and the daily LT3 dose added renders serum T3 levels within the middle to upper normal range.

### Working Hypothesis 2

Success with combination therapy depends strongly on a patient's RTF as well as the LT3 added to sufficient LT4 dosing. Little or no success is predicted when RTF is 20% or more unless the daily LT3 dose added generates serum T3 levels in the mid-normal to high normal T3 range. Preference for combination therapy is not likely unless the added T3 generates high-normal range to supra-physiologic T3 levels.

## Methods

### Dosage Response Simulations

**Figure 1:**
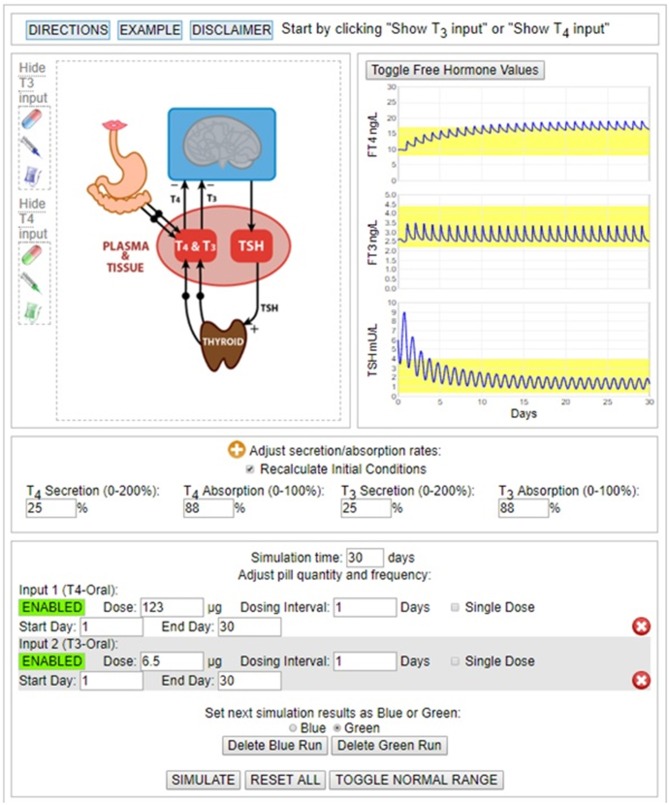
Simulation of 123 μg LT4 + 6.5 μg LT3 dosing experiment in Siegmund 2004 trial (20). Final hormone values achieved with ~25% RTF.

The THYROSIM app (27) has been applied in the current work by exploring THYROSIM responses to exogenous LT4 and combination LT4 + LT3 hormone dosage inputs in simulated patients with primary hypothyroidism, and patients with different degrees of RTF, rendered hypothyroid by autoimmune thyroid disease, radioactive iodine therapy, external beam radiotherapy, or thyroid surgery. In support of predicted results, simulation conditions—namely dosages and predicted RTF—were adjusted to and compared with data from several studies of patients receiving synthetic combination LT4 + LT3 therapy in comparison with LT4 therapy alone (9–22). An example of a simulation matching data from Siegmund et al. (20) is shown in Figure 1.

### RTF Measures

To obtain RTF estimates for our data with the THYROSIM app, we simulated patient dosing input regimens and output responses with serum TSH, T4, and T3 presumed to be measured before any therapy was begun. RTF is estimated by manually adjusting the T4 and T3 secretion rates on the graphic interface of the THYROSIM app, by trial-and-error. The goal is to find the best RTF (% secretion rates) that generates starting values (initial hormone concentrations) that approximate both the initial T4, T3, and TSH concentrations measured prior to dosing therapy (combination therapy or T4 monotherapy), and the approximate final concentrations measured at the end of the study period.

For hypothyroid patients with different etiologies of their hypothyroidism, it is important that these thyroid variables are assessed after they reach a steady state, after they plateau, and after the degree of RTF also stabilizes. For example, following complete thyroidectomy, the thyroid hormone and TSH levels 6 weeks later should indicate 0% RTF. For someone with Hashimoto's hypothyroidism, in order to predict their likely non-zero RTF, at least 6 weeks are needed for the thyroid hormone and TSH levels to stabilize following likely incomplete thyroid destruction.

Only one study was available from among the 14 combination therapy trials that provided any measured patient hormone values prior to initiating therapy for hypothyroidism, and this was only for TSH (16). THYROSIM simulations were conducted with different RTF values, by varying the thyroidal T4 and T3 secretion rates from 0 (athyreotic) to 50% and recording the starting values for total serum TSH, T4 and T3. We assumed that T3 and T4 secretion rates (adjustable on the THYROSIM interface) are suppressed or reduced together by relatively the same amounts.

## Results

### Method Validation

**Figure 2:**
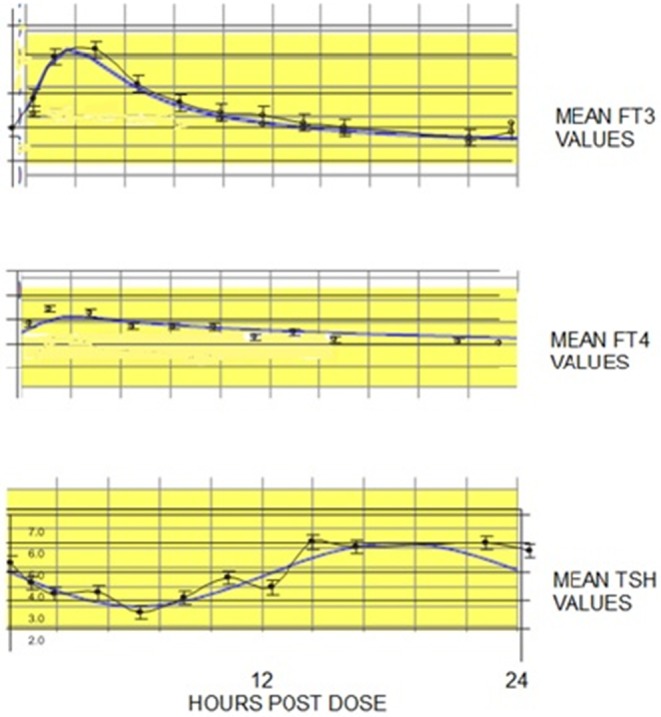
FT3, FT4, and TSH 24 h temporal responses (blue curves) predicted by THYROSIM for simulated 117 μg T4 + 10 μg T3 dosing, superimposed over corresponding LT3, LT4, and TSH data (black dots with error bars) collected over 24 h, in 10 patients from a larger study in hypothyroid patients on combined LT3/LT4 therapy (18–34). Yellow bands represent normal ranges.

To help validate our computational modeling approach, we simulated the combination therapy dosing and dose-response conditions reported in the study of 10 patients from the Saravanan trial, which provided 24 h hormone profiles of TSH, FT3 and FT4 in 20 hypothyroid patients taking either LT4 monotherapy or combined LT3/LT4 therapy (34). Simulation response results (solid blue lines) are shown graphically in Figure 2, superimposed over the published data corresponding to these results. They match the data quite well. In particular, the ~40% rise in mean FT3 values, peaking at ~4 h, is well represented by the simulation and is shown to remain within the normal FT3 range (yellow band), thus tracking the previously reported data well. In comparison, the Saravanan sub-study (34) reported 3 of 10 patients in the LT3/LT4 group, but none in the LT4 alone group, had FT3 levels above their laboratory reference range at some time over the 24-h period, but lasting only for <2 h.

### Addressing the Hypotheses

A summary of conditions, patient populations and hypothyroidism etiologies from 13 combination therapy trials (excluding Valizadeh) is given in Table 1. This table shows (where available) the LT4 doses prior to randomization, and the LT4 and LT4/LT3 doses in the monotherapy and combination therapy arms. Full information about the etiology of the hypothyroidism was not provided in all trials.

**Table 1:** Summary of 13 trials of synthetic combination with LT4/LT3 therapy compared to LT4 alone.

**References**	**Treatment dosing**	**Dose of LT4 pre-trial**	**Dose of LT4 in LT4 gp (number of patients)**	**Dose of LT4 in LT3 gp (number of patients)**	**Dose of LT3 in LT3 gp**	**Etiology primary hypo-thyroidism (number of patients)**	**Design**	**Number of patients randomized (completed follow-up)**	**Treatment duration**	**Baseline & end of study TSH differences between groups**
Appelhof et al. (9)	T4: usual dose LT4/LT3: 10:1 or 5:1 ratio of T4 to T3 ratio, respectively Dosing: Twice daily for both LT4 & LT3	1.46 μg/kg/day (placebo) 1.61 μg/kg/day (LT4: LT3 10:1) 1.73 μg/kg/day (LT4: LT3 5:1)	100 μg (50 μg given twice daily)	75 μg (10:1 ratio) 75 μg (5:1 ratio) (approx. 37.5 μg given twice daily)	7.5 μg (3.75 μg twice daily) (10:1) 15 μg 7.5 μg twice daily (5:1)	Autoimmune (other causes excluded), 80% positive TPO antibodies	Parallel, blinded	141 (130)	15 weeks	Baseline TSH values 1–1.1. LT4 vs. LT4/LT3 (10:1) vs. LT4/LT3 (5:1) 0.64 vs. 0.35 vs. 0.07 (TSH lower in the 5:1 T3:T4 dose group)
Bunevicius et al. (11)	T4: usual LT4/LT3: usual T4 dose minus 50 μg/day with T3 12.5 μg/day Dosing: Once daily	175 μg (all) 181 μg (placebo first) 169 μg (LT3 first)	175 μg	125 μg	12.5 μg	Mixed—Autoimmune (16), thyroid cancer (17)	Cross-over, blinded	35 (33)	5 weeks	Baseline TSH 0.3–1.5. LT4 0.8 vs. LT4/LT3 0.5. NS‡ difference
Bunevicius et al. (10)	T4: usual LT4/LT3: usual T4 dose minus 50 μg/day with T3 10 μg/day Dosing: Once daily	All: 100 μg (7) 150 μg (3)	115 μg (approx.)	65 μg (approx.)	10 μg	All Graves disease, history of subtotal thyroidectomy	Cross-over, blinded	13 (10)	5 weeks	Baseline TSH 1.02. LT4 0.45 vs. LT4/LT3 0.47. NS‡ difference
Clyde et al. (27)	T4: usual LT4/LT3: usual T4 dose minus 50 μg/day with T3 15 μg/d Dosing: Twice daily LT3, LT4 once daily	131 μg (placebo) 136 μg (LT3) 1.6 μg/kg/day (placebo) 1.8 μg/kg/day (LT3)	131 μg (including 25 μg BID, balance given once daily)	86 μg once daily	15 μg (7.5 μg twice daily)	Mixed – Autoimmune (31), post-RAI* (10), thyroid surgery (1), post-EBRT**(1), thyroid cancer (1)	Parallel, blinded	46 (44)	4 months	Baseline TSH 2.2–2.6. LT4 2.1 vs. LT4/LT3 2.0. NS‡ difference
Escobar-Morreale et al. (28)	T4: 100 μg/day LT4/LT3: LT4 75 μg/day and T3 5 μg/d Dosing: Once daily	100 μg (all)	100 μg	75 μg 87.5 μg (add on)	5 μg 7.5 μg (add on)	Mixed – Autoimmune (23), post-RAI* (5)	Cross-over, blinded	28 (26)	8 weeks	Baseline TSH “normal”. LT4 1.95 vs. LT4/LT3 2.56. LT4/LT3 > LT4
Fadeyev et al. (29)	T4: 1.6 μg/kg/day LT4/LT3: estimated T4 dose minus 25 μg/day with T3 12.5 μg/day Dosing: Once daily^†^	50–125 μg (?)	100 μg (25) 125 μg (7) 75 μg (9) 50 μg (1)	75 μg (median) 75 μg (10) 100 μg (4) 50 μg (2)	12.5 μg	All autoimmune	Parallel, unblinded	58 (58?)	6 months	Baseline TSH “normal”. LT4 1.35 vs. LT4/LT3 1.7. NS‡ difference
Kaminski et al. (30)	T4: 125 or 150 μg LT4/LT3 75 μg + 15 μg T3 Once daily	125 or 150 μg	125 or 150 μg	75 μg	15 μg	Mixed – Autoimmune (23), post-RAI* (3), thyroid cancer (6)	Cross-over, blinded	32	8 weeks	Baseline TSH 0.31. LT4 0.19 vs. LT4/LT3 0.64 NS‡ difference
Nygaard et al. (31)	T4: usual LT4/LT3: usual T4 dose minus 50 μg/day with T3 20 or 50 μg/day, respectively Dosing: Once daily	129 μg (all)	131 μg	77 μg	20 μg	Autoimmune (all positive TPO antibodies)	Cross-over, blinded	68 (59)	12 weeks	Median TSH at diagnosis 43–82, baseline TSH 1.1, LT4 0.99 vs. LT4/LT3 0.76. NS‡ difference
Rodriguez et al. (32)	T4: usual LT4/LT3: usual T4 dose minus 50 μg/day with T3 10 μg/day Dosing: Once daily^†^	121 μg (all) 118 μg (seq1, placebo) 121 μg (seq2, LT3)	118 μg	121–50 μg = 71 μg	10 μg	Mixed—Autoimmune (23), post-RAI* (4), thyroid surgery (3)	Cross-over, blinded	30 (27)	6 weeks	Baseline TSH 1.7-1.8. LT4 2.5–2.9 vs. LT4/LT3 3.3–7.6. NS‡ difference
Saravanan et al. (33)	T4: usual LT4/LT3: usual T4 dose minus 50 μg/day with T3 10 μg/day Dosing: Once daily	123 μg (placebo) 127 μg (LT3)	123 μg	127–50 μg = 77 μg	10 μg	Primary hypothyroidism (72%?, 44% TPO antibodies), no thyroid cancer	Parallel, blinded	697 (573)	12 Months (outcomes assessed 3 and 12 months)	Baseline TSH 0.84-0.85. LT4 0.79 vs. LT4/LT3 1.25 at 12 months. LT4/LT3 > LT4 at 3 months
Sawka et al. (34)	T4: usual LT4/LT3: 50% usual T4 dose with T3 total 25 μg/day (12.5 μg BID) Dosing: Twice daily T3, once daily T4	120 μg (placebo) 132 μg (LT3)	118 μg	67 μg	19 μg (9.5 μg twice daily)	Primary hypothyroidism, excluded: thyroid cancer, history of hyperthyroidism, thyroidectomy	Parallel, blinded	40 (33)	15 weeks	Baseline TSH 1.75-2.2. LT4 1.7 vs. LT4/LT3 1.8. NS‡ difference
Siegmund et al. (35)	T4: usual LT4/LT3: usual T4 dose minus 5% with T3 5% (aim 14:1 ratio LT4 to T3) Dosing: Once daily^†^	100 μg (5) 125 μg (12) 150 μg (8) 175 μg (1)	129 μg	123 μg	6.5 μg	Mixed – Autoimmune (2), post-RAI* or thyroid surgery (24)	Cross-over, blinded	26 (23)	12 weeks	Baseline TSH 1.72. LT4 1.5 vs LT4/LT3 0.5. LT4/LT3 < LT4
Walsh et al. (37)	T4: usual LT4/LT3: usual T4 dose minus 50 μg/day with T3 10 μg/day Dosing: Once daily^†^	136 μg	136 μg	86 μg	10 μg	Mixed—Autoimmune (94), post-RAI* (4), thyroid surgery (12), no thyroid cancer	Cross-over, blinded	110 (101)	10 weeks	Baseline TSH 1.3-1.5. LT4 1.5 vs. LT4/LT3 3.1. LT4/LT3 > LT4

† *Dosing not reported, assume once daily*.

### Predicted TSH, T4, and T3 Levels vs. RTF Values at Diagnosis

**Figure 3:**
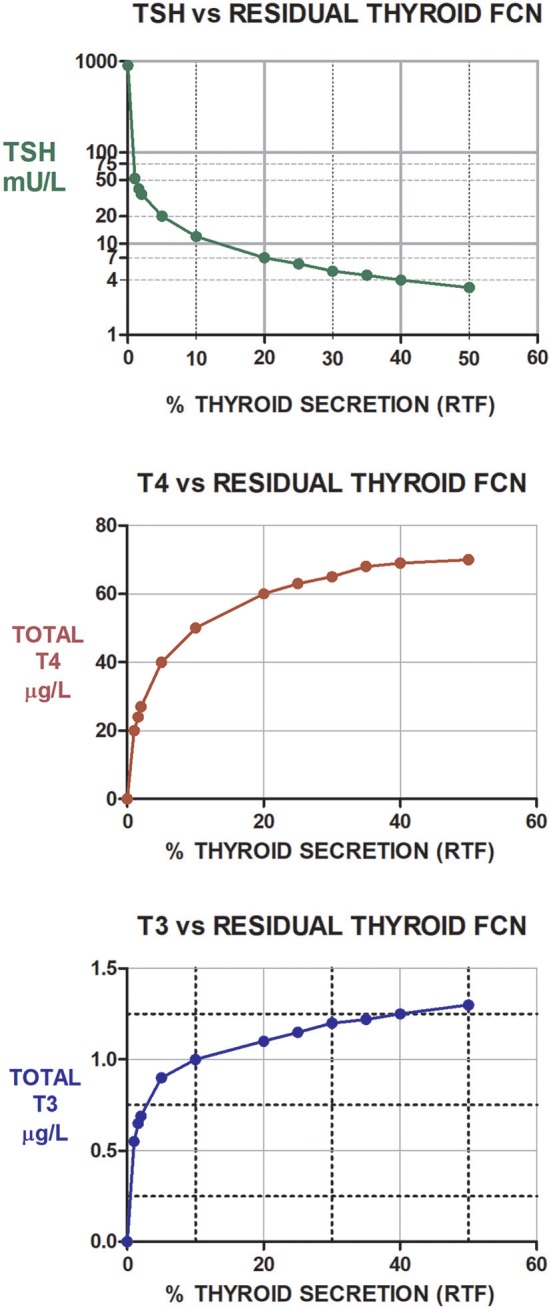
Illustration of the predicted (and non-linear) TSH, T4, and T3 levels prior to initiating any therapy for hypothyroidism in individuals with RTFs varying between 0% (athyreotic) up to 50% RTF (hemi-thyroidectomized). Notably, as RTF increases from about 1–10%, TSH levels fall by ~80%, while T4 levels increase by ~163% and T3 levels increase by ~79%. Similarly, T4 increases by ~30%, T3 by 20% and TSH falls about 58% as RTF increases from 10 to 30%. TSH levels shown in green, T4 levels shown in red, and T3 levels shown in blue.

The three graphs shown in Figure 3 illustrate the predicted TSH, T4, and T3 levels prior to initiating any therapy for hypothyroidism in individuals with RTF varying between 0% (athyreotic) up to 50% RTF. The relationships are nonlinear, particularly in the most likely RTF range, up to 30%; and TSH—followed by T4–followed by T3 levels, are the most sensitive to increasing RTF.

### RTF Estimates and Predicted Success of LT3/LT3 Therapy

#### Patient RTF Values

The best results predicted by THYROSIM and supported by the trial data suggest that, because the trials included patients with different etiologies of hypothyroidism, the participants had varying degrees of RTF. Table 2 shows the various trials separated into categories of simulated high, medium and low RTF values, respectively. The data in published results were incomplete, so the categories may not be completely accurate.

**Table 2:** Measured mean TSH, FT4, T4, T3, FT3 values at beginning and end of trials for monotherapy vs. combination therapy groups in trials grouped according to whether patients were estimated to have high RTF (>20%), medium RTF (10–20%), or low RTF (<10%).

**Trial**	**TSH before**	**TSH end**	**FT4 (ng/dl) before**	**FT4 end**	**T3 (ng/dl) before**	**T3 end**	**FT3 (pg/dl) before**	**FT3 end**	**T3 &** T4 **ranges by sim**	**LT3 dose (μg)**	**LT4 dose (μg)**
**High RTF (>20%)**											
Appelhof T4	1.0	0.64	1.15	1.18	111	111	–	–	–		
Appelhof T4 + T3 (10:1)	1.1	0.35	1.15	1.02	109	119	–	–	Med Med	7.5	75
Appelhof T4 + T3 (5:1)	1.0	0.07	1.18	1.00	115	143	–	–	Med-HighMed	12.5	75
Bunevicius, 2002, T4	1.02	0.45	1.61	1.64	–	227	–	–	–		
Bunevicius, 2002, T4 + T3	1.02	0.47	1.61	0.96	–	247	–	–	High High	10	65
Escobar-Morreale T4	nl	1.95	–	1.61	–	–	–	332	–		
Escobar-Morreale T4 + T3 (5 μg)	nl	2.56	–	1.31	–	–	–	325	Med Med	5	75
Escobar-Morreale T4 + T3 (7.5 μg)	nl	1.09	–	1.34	–	–	–	384	Med Med	7.5	87.5
Siegmund T4	1.72	1.5	1.72	1.62	–	–	332	294	-		
Siegmund T4 + T3	1.72	0.5	1.72	1.56	–	–	332	324	Low- Med High	6.5	123
**Medium RTF (10–20%)**											
Clyde T4	2.2	2.1	1.2	1.2	96	87	–	–	–		
Clyde T4 + T3	2.6	2.0	1.3	0.8	89	135	–	–	Med Med	15	86
Fadeyev T4	–	1.35	–	1.45	–	–	–	273			
Fadeyev T4 + T3	–	1.7	–	0.96	–	–	–	267	Med Med	12.5	75
Kaminski T4	0.31	0.19	1.26	1.64	93	103	–	–	–		
Kaminski T4 + T3	0.31	0.64	1.26	1.03	93	98	–	–	Med -High Med	15	75
Sawka T4	2.2	1.7	1.30	1.38	–	–	280	286	–		
Sawka T4 + T3	1.75	1.8	1.22	0.82	–	–	267	306	Med *Low*	19	67
Rodriguez T4	1.7–1.8	2.5–2.9	11–11.2	10.8–10.9	76–79	73–86	–	–	–		
Rodriguez T4 + T3	1.7–1.8	3.3–7.6	11–11.2	7.6–8.2	76–79	95–104	–	–	Med Med	10	71
**Low RTF (<10%)**											
Bunevicius, 1999, T4	0.3	0.8	2.0	2.3/*15.2*	–	87	–	–	–		
Bunevicius, 1999, T4 + T3	1.	0.5	1.9	1.8/*11.3*	–	117	–	–	High Med-High	12.5	125
Nygaard^*^ T4	1.1	0.99	–	–	–	–	–	–	–		
Nygaard^*^ T4+T3	1.1	0.76	–	–	–	–	–	–	High *Low*	20	77
Saravanan T4	0.87	0.79	1.62	1.57	–	–	248	234	-		
Saravanan T4 + T3	0.85	1.25	1.64	1.14	–	–	248	239	Med Med-High	10	77
Walsh T4	1.4	1.5	1.19	1.21	–	–	221	241	-		
Walsh T4 + T3	1.4	3.1	1.19	0.89	–	–	221	228	Low-Med Med	10	86

*Gray shading indicates T4/T3 arm of study*,

* *study reports only free T4 index and FT3 index and does not report either total or free T4 or T3, blue font is total T4 levels in mcg/dL*.

*“T3 and T4 levels by sim” are the levels predicted by simulation for the combination therapy group, rather than measured T3 and T4 levels, and are categorized into 3 groups (high/medium/low). Mean/median LT3 and LT4 doses in the combination therapy group are also shown*.

For the first category of high RTF (>20%), no benefit of combination therapy was predicted with respect to quality of life, mood or neurocognitive benefit or LT4/LT3 preference in the 4 trials with high RTFs (9, 10, 13, 20). We predicted that with >20% RTF, small amounts of added LT3 would have less of an impact, as assessed by various outcome measures or patient preference. We speculated that combination therapy would only be clinically successful in the setting of high RTF if the LT4 dose maintained T4 and TSH in their normal ranges and the added LT3 dose generated higher than normal T3 levels. For higher LT3 doses, the effect became noticeable and combination therapy was more likely to be preferred, albeit potentially toxic.

For the second category of medium RTF (10–20%), some benefit with respect to quality of life or mood or neurocognitive benefit was predicted in the five relevant trials (12, 14, 15, 17, 19). However, the impact of the modest amount of added LT3 on outcome measures was expected to be minimized by the endogenous RTF. If RTF was low (<10%), combination therapy was predicted to provide substantial quality of life or mood or neurocognitive benefit and/or to be preferred by patients in the 4 relevant trials (11, 16, 18, 22).

#### Successful Therapy Based on Improved Outcome Measures

**Figure 4:**
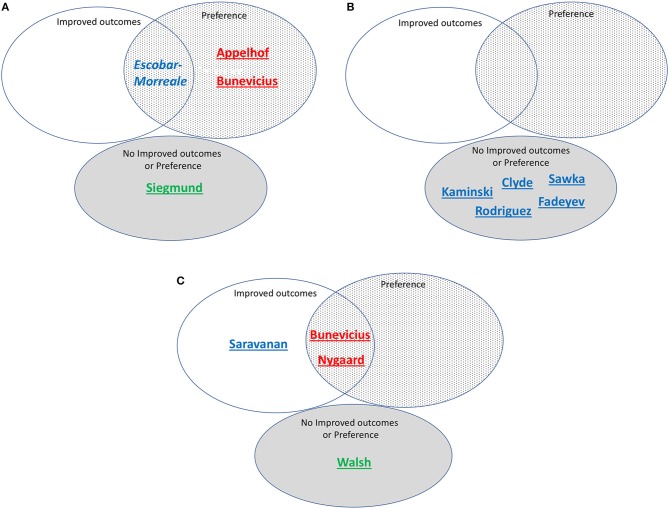
**(A)** Studies assessed as including patients with high residual thyroid function (>20%) and/or T3 levels predicted by simulation, grouped according to whether improved outcomes, preference, both improved outcomes and preference, or neither improved outcomes nor preference was reported. Red font indicates high simulated T3 levels. Blue font indicates medium simulated T3 levels. Green font indicates low simulated T3 levels. Underlined indicates correctly predicted. Italics indicate incorrect prediction. Bunevicius is 2002 study. **(B)** Studies assessed as including patients with medium residual thyroid function (10-20%) and/or T3 levels predicted by simulation, grouped according to whether improved outcomes, preference, both improved outcomes and preference, or neither improved outcomes nor preference was reported. Red font indicates high simulated T3 levels. Blue font indicates medium simulated T3 levels. Underlined indicates correctly predicted. **(C)** Studies assessed as including patients with low residual thyroid function (<10%) and/or T3 levels predicted by simulation, grouped according to whether improved outcomes, preference, both improved outcomes and preference, or neither improved outcomes nor preference was reported. Red font indicates high simulated T3 levels. Blue font indicates medium simulated T3 levels. Green font indicates low simulated T3 levels. Underlined indicates correctly predicted. Bunevicius is 1999 study.

Table 3 shows the same 13 trials separated into three categories: (a) those showing substantial improvement in outcomes with combination therapy (11, 16); (b) those showing partial benefit based on some outcome measures, but not others (13, 18); and (c) those showing no benefit (9, 12, 14, 15, 17, 19, 20, 22). These same trials are also shown in Figures 4A–C, which categorize the trials by RTF and show the associated trial results displayed as Venn diagrams.

**Table 3:** Thirteen trials of monotherapy vs. combination therapy, categorized according to whether patients experienced benefits or not during combination therapy.

**Benefit as assessed by improved outcomes**
**a) Substantial quality of life or mood or neurocognitive benefit**
Bunevicius, 1999
Nygaard
**b) Some quality of life or mood or neurocognitive benefit**
Escobar-Morreale (5 μg T3)
Escobar-Morreale (7.5 μg T3)
Saravanan^*^
**c) No quality of life or mood or neurocognitive benefit**
Appelhof (T4 + T3, 10:1 ratio)
Appelhof (T4 + T3, 5:1 ratio)
Bunevicius, 2002
Clyde
Fadeyev
Kaminski
Rodriguez
Sawka
Siegmund
Walsh

* *Showed benefit at 6 months but not at 12 months*.

#### Successful Therapy Based on Treatment Preference

Treatment preference was assessed in 7 of the 13 trials. Table 4 lists these trials in two categories: (a) trials in which a preference for combination therapy was expressed by participating patients (9–11, 13, 16); and (b) trials in which there was no patient preference for combination therapy (18, 22). These trials are also indicated in Figures 4A–C, showing how preference is related to degree of RTF, and whether either improved outcomes, preference, or both improved outcomes and preference were demonstrated in the same trial. Additionally, indicated on the figure is whether the T3 levels were predicted to be low, medium or high during the trial.

**Table 4:** Seven trials of monotherapy vs. combination therapy, categorized according to whether patients preferred combination therapy or not.

**Therapeutic preference**
**a) Preference for combination therapy**
Appelhof (T4 + T3, 10:1 ratio)
Appelhof (T4 + T3, 5:1 ratio)
Bunevicius, 1999
Bunevicius, 2002
Escobar-Morreale (5 μg T3)
Escobar-Morreale (7.5 μg T3)
Nygaard
**b) No preference for combination therapy**
Saravanan
Walsh

### Testing Hypothesis 1

For our first hypothesis that achievement of medium-high T3 levels along with sufficient LT4 in the dose is needed for successful (improved outcomes or preference) combination therapy, our prediction was mostly correct. The Appelhof, Bunevicius, and Nygaard studies (9–11, 16) were predicted to have high T3 levels and were “successful” (see Figures 4A,C). The Escobar-Morreale, Kaminski, Clyde, Sawka, Rodriguez, Saravanan, and Fadeyev trials (12–15, 17–19) (Figures 4A–C) were predicted to have medium T3 levels and therefore the RTF might also impact their success. The Walsh and Siegmund trials (20, 22) (Figures 4A,C) were predicted to have low or low medium T3 levels and did not report improved outcomes or preference.

### Testing Hypothesis 2

With respect to our second hypothesis of the degree of RTF (while also taking the T3 levels achieved into account) affecting the success of combination therapy, results of this prediction are shown in Figure 4. Figure 4A shows the studies with high RTF and three out of four studies are correctly predicted as not showing combination therapy to be successful. Figure 4B shows the studies with medium RTF and all five studies are correctly predicted as not showing combination therapy to be successful. For the prediction that low RTF would be associated with successful combination therapy due to the more noticeable effect of the added LT3, we showed in Figure 4C that four out of four studies were correctly predicted in this category.

### Recommendations for Combination Therapy Dosing in Patients Previously Untreated With T4 or T3

**Figure 5:**
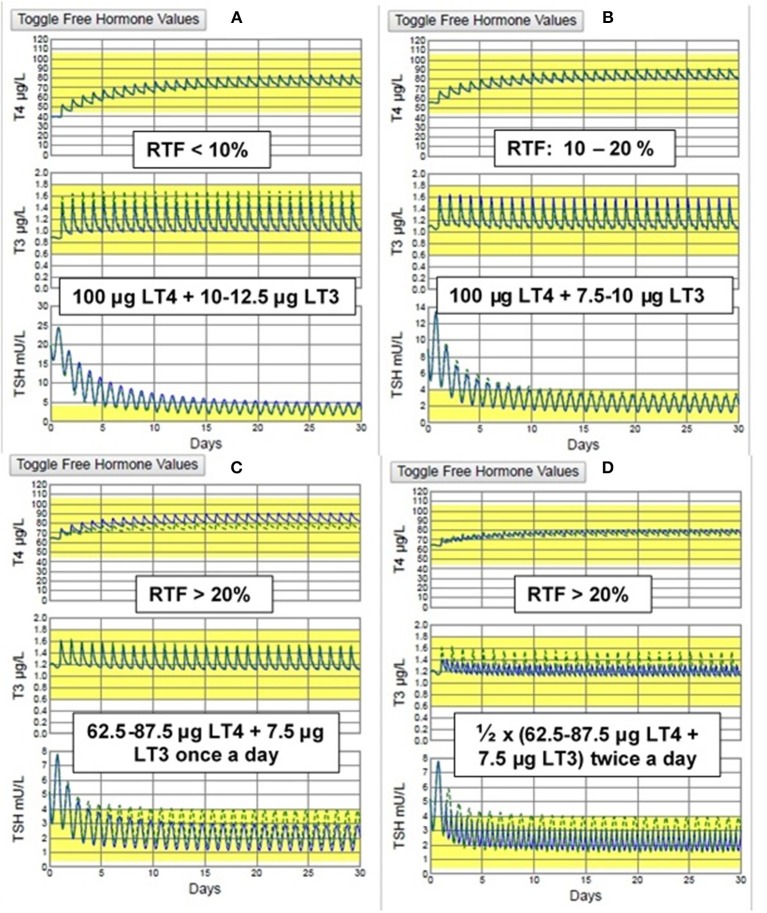
THYROSIM simulated T4, T3, & TSH responses to the recommended dosage combinations: for **(A)** low RTF (<10%) & **(B)** medium RTF (10–20%) (TOP) & **(C)** high (>20%) RTF using once daily dosing and **(D)** high (>20%) RTF using twice a day dosing. (BOTTOM). The smallest T3 (and TSH) excursions are seen with twice daily dosing, but no values are outside the normal ranges with once a day dosing.

Our estimates of RTF allow us to make predictions regarding the dosing of LT3 that should be optimal when designing a combination therapy trial. Serum levels of TSH, T4 and/or T3 should be obtained at the time of diagnosis, either from patient history data or anew, prior to initiating any therapy and one or more (preferably >1) of the graphs in Figure 3 can then be used as nomograms to estimate RTF. We would then predict that the following practical daily dosing combinations would serve best for starting dosing in 70 kg individuals with computed RTFs in the three given ranges. (These recommended dosages should be adjusted for body weight or other anthropomorphic measures.) To maximize compliance, once-a-day dosing responses are simulated in Figures 5A–C for <10% RTF, 10–20% RTF, and >20% RTF. This should keep T3 excursions within the normal range, as shown in the figures. If individual patient clinical requirements warrant, the LT4 + LT3 dosages can be split in half and prescribed 2x a day, with smaller excursions in serum T3, as shown in Figure 5D.

$$\begin{array}{l} \text{ For RTFs}<10\%\to 100\;\mu \text{g LT}4+10-12.5\;\mu \text{g LT}3,\text{oncedaily}\\ \text{For RTFs }10-20\%\to 100\;\mu \text{g LT}4+7.5-10\;\mu \text{g LT}3,\text{oncedaily}\\ \text{ For RTFs}>20\%\to \text{LT}4=87.5\text{ ug}+7.5\text{ ug LT}3,\text{oncedaily}\\ \text{ For RTFs}>20\%\to \text{LT}4=50\%\text{ of }87.5\text{ ug}\\ \;+\;50\%\text{ of }7.5\text{ ug LT}3,\text{twicedaily}. \end{array}$$

## Discussion

Our two working hypotheses are reasonably well-supported by our simulation data and comparative analysis with the data from the 13 combination trials. Evidently, these hypotheses are strongly intertwined, in a complex way, probably as a consequence of the tight, nonlinear couplings and homeostatic feedback effects among these well-regulated hormones. Importantly, the T3 (and T4) levels that can be achieved during combination therapy, and whether they are low, medium or high, appear to be affected endogenously by RTF—in a nonlinear way—as well as by the exogenous LT4 and LT3 dosages given. The latter are immediately under the influence of the same endogenous regulatory system components following absorption of the dosages. Overall, if the RTF is low, the added T3 seems to provide more impact in terms of either improved outcomes or patient preference. If the RTF is high, the same dose of T3 appears to have less impact However, if the amount of T3 added is relatively high, thus achieving a high or supraphysiologic T3 level, then there also is a positive impact in terms of either improved outcomes or patient preference—with due consideration to the clinical effects of T3 toxicity.

We recognized in existing trial data that, in the presence of sufficient T4, the T3 levels needed to ensure patient preference were higher than those needed to provide improved outcome measures; and this was borne out by our analyses. This motivated our coupled hypotheses and their analysis by “what-if” simulations of the trial data. We found a similar number of studies (five studies) associated with patient preference for combination therapy (9–11, 13, 16) as those demonstrating improved outcomes (four studies) (11, 13, 16, 18). Four of the five studies that showed patient preference had high simulated (and measured) T3 levels (9–11, 16), the exception being the Escobar-Morreale study (13) in which the simulated T3 levels were mid-range.

Our analysis was limited by several complicating factors present or absent in the trial data. T4/T3 ratios reported in the various studies were very different, some with initially higher T4/T3 ratios at baseline and the T4/T3 ratios substantially lower in the combination therapy arm (15, 17). In addition, not all studies provided full laboratory values at baseline, during the study, or at the end of the study [e.g., Nygaard (16)]. In a few studies dosing regimens were not clear. One study (18) showed improved outcomes at 6 months, but not at 12 months. We classified this study as having a positive outcome, in part because all other studies were 6 months or less in duration. However, this may not be the best way to categorize this trial, which may have demonstrated a placebo effect at 6 months. The 2002 Bunevicius study (6) was not amenable to comparative analysis as the RTF appeared to be >100%; this might be because this hypothyroid trial population consisted of Graves' disease patients who had undergone surgery for their disease, which may have been incomplete, with enough residual thyroid tissue to make dosing formulation more difficult. The Valizadeh study (21) could not be simulated for unclear reasons.

Additional limitations of prior studies that might have affected the rigor of our analyses include the following. The studies clearly included patients with different etiologies of their hypothyroidism and a wide spectrum of RTF values. There is inter-assay variability across the various studies conducted in various countries, especially for FT3 assays, making it difficult to obtain very close comparative results in all cases. Not all studies reported the timing of phlebotomy, and whether blood samples were drawn at random times of day or were trough levels, making it possible that thyroid hormone levels, particularly T3 or FT3 could vary by as much as 40% (34).

There are also limitations of the trials that, in turn, may have led to limitations in our analysis of them. With regard to patient satisfaction and patient preference issues assessed in the various trials, it must be acknowledged that many symptoms of hypothyroidism are non-specific and overlap with symptoms of other conditions (35). Therefore, it is possible that lack of improvement in quality of life, mood, or neurocognitive function noted could have occurred because reported deficits were not thyroid-related. It is also possible that improvements in mood or preference for combination therapy were reported because a different condition such as depression was in fact being treated (36, 37). In addition, some of these trials may have been too short to allow sufficient adaptation for either benefits to be seen or adverse effects to occur. Despite these limitations, we believe we have achieved the goal of our studies.

In summary, our results reliably support the notion that RTF differences are a key factor in explaining the ambiguities in the spectrum of combination therapy study results reported between 1999 and 2016. As added value, we have adapted our RTF estimation methodology for combined LT4 + LT3 dosing that is practical and potentially optimal when designing a combination therapy trial. Serum TSH, T4 and/or T3 levels at the time of diagnosis should be obtained from patient history data or anew, and prior to initiating any therapy; and one or more of the three graphs in Figure 3 can then be used as nomograms to estimate RTF from individual patient data. Using this algorithm, we have provided combination dosing schemes that should serve best for starting dosing in 70 kg individuals with computed RTFs in the three given ranges. These are readily scaled by individual patient requirements, body weights or other anthropomorphic measurements.

## Data Availability Statement

The datasets generated for this study are available on request to the corresponding author.

## Author Contributions

All authors listed have made a substantial, direct and intellectual contribution to the work, and approved it for publication.

### Conflict of Interest

JD is the creator and developer of the THYROSIM app. The remaining author declares that the research was conducted in the absence of any commercial or financial relationships that could be construed as a potential conflict of interest.
